# New insights into the stemness of adoptively transferred T cells by γc family cytokines

**DOI:** 10.1186/s12964-023-01354-3

**Published:** 2023-12-04

**Authors:** Mengshi Luo, Wenjian Gong, Yuewen Zhang, Huayi Li, Ding Ma, Kongming Wu, Qinglei Gao, Yong Fang

**Affiliations:** 1 Department of Gynecological Oncology, Tongji Hospital, Tongji Medical College, Huazhong University of Science and Technology, Wuhan, China; 2 National Clinical Research Center for Obstetrics and Gynecology, Cancer Biology Research Center (Key Laboratory of the Ministry of Education), Tongji Hospital, Tongji Medical College, Huazhong University of Science and Technology, Wuhan, China; 3 Department of Oncology, Tongji Hospital of Tongji Medical College, Huazhong University of Science and Technology, Wuhan, China

**Keywords:** Stemness, Stem cell-like memory T cells (T_SCM_), Adoptive cell therapy (ACT), γc family cytokines

## Abstract

T cell-based adoptive cell therapy (ACT) has exhibited excellent antitumoral efficacy exemplified by the clinical breakthrough of chimeric antigen receptor therapy (CAR-T) in hematologic malignancies. It relies on the pool of functional T cells to retain the developmental potential to serially kill targeted cells. However, failure in the continuous supply and persistence of functional T cells has been recognized as a critical barrier to sustainable responses. Conferring stemness on infused T cells, yielding stem cell-like memory T cells (T_SCM_) characterized by constant self-renewal and multilineage differentiation similar to pluripotent stem cells, is indeed necessary and promising for enhancing T cell function and sustaining antitumor immunity. Therefore, it is crucial to identify T_SCM_ cell induction regulators and acquire more T_SCM_ cells as resource cells during production and after infusion to improve antitumoral efficacy. Recently, four common cytokine receptor γ chain (γc) family cytokines, encompassing interleukin-2 (IL-2), IL-7, IL-15, and IL-21, have been widely used in the development of long-lived adoptively transferred T_SCM_ in vitro. However, challenges, including their non-specific toxicities and off-target effects, have led to substantial efforts for the development of engineered versions to unleash their full potential in the induction and maintenance of T cell stemness in ACT. In this review, we summarize the roles of the four γc family cytokines in the orchestration of adoptively transferred T cell stemness, introduce their engineered versions that modulate T_SCM_ cell formation and demonstrate the potential of their various combinations.

Video Abstract

**Supplementary Information:**

The online version contains supplementary material available at 10.1186/s12964-023-01354-3.

## Background

 Cancer is the leading cause of death globally and a predominant obstacle to increasing life expectancy [1, 2]. The emergence of immunotherapy has revolutionized cancer treatment and offers more treatment options for patients with cancer [3, 4]. Tumor immunotherapy mainly consists of immune checkpoint blockade (ICB) and adoptive T-cell therapy (ACT). ACT, including chimeric antigen receptor therapy (CAR-T) and engineered T-cell receptor T cell therapy (TCR-T), relies on targeted destruction of cancer cells by potent antitumor T cells associated with the CD8(+) T cell state [5–9]. Despite the substantial antitumor activity in hematological tumors, adoptively transferred CAR-T cells have a limited effect in solid tumors, mainly due to poor expansion and persistence in vivo [8, 10–16]. During chronic stimulation of tumor antigens, adoptively transferred T cells are inevitably exhausted and exhibit an anergic state of cytotoxicity loss by virtue of the progressive expression of co-inhibitory molecules such as PD-1, TIM-3, LAG-3, CTLA-4, and TIGIT [6, 13]. As a result, approaches to acquiring long-lived functional T cells with stem cell-like properties, termed stem cell-like memory T cells (T_SCM_), should be urgently developed for CAR-T therapy.

T cell stemness is termed to describe the stem cell-like behavior of T cells, including self-renewal, multipotency, and functional persistence. T_SCM_ cells, featured by CD45RA^+^ CD45RO^−^ CD27^+^ CD28^+^ CCR7^+^ CD62L^+^ CD95^+^ CD122^+^ CD127^+^, were first discovered in mouse models of human graft versus host disease (GVHD) in 2005 and isolated in vitro in 2013 [5, 17–21]. As a unique subset of memory T cells, apart from memory traits, T_SCM_ cells receive the stem cell-like attributes, that are the self-renewal and multipotent ability to continually generate all memory and effector T cell subsets. To identify T_SCM_ cells with analogous properties, Gattinoni et al. stimulated naïve T cells by triggering Wnt signaling with Wnt3A or inhibitors of glycogen synthase kinase-3β (GSK-3β) TWS119 [20, 21].

At present, steady progress has been made with respect to the induction of T cell stemness. Of note, many factors involved in the generation and maintenance of T_SCM_ cells are known, such as Notch [22–24], Wnt [25–27], mTOR [28], and cGAS-STING [29] signaling pathways, cytokines, and transcriptional factor c-Myb [5, 30, 31]. Thereinto, cytokines like IL-7 and IL-15 were listed among the top twelve immunotherapeutic agents with wide appeal to the immunotherapy and, by consensus, held particular promise for use in cancer therapy, as shown by the US National Cancer Institute in 2008 [32]. With the emergence of cytokine therapy for cancer, the four cytokines of the common cytokine receptor γ chain (γc, CD132) family, containing interleukin-2 (IL-2), IL-7, IL-15, and IL-21 are dictated to regulate the T cell stemness formation and maintenance. They serve as the third signal that triggers the antigen-specific immunological response and are theoretically demonstrated as essential factors to coordinate the differentiation and the cytotoxicity of CD8(+) T cells via the formation of the tight immunological synapse [33]. Thus, the cytokine milieu plays a fate-defining role for T cells. The four γc family cytokines alone or their different combination may substantially affect the modulation of adoptively transferred T cell stemness. Applying these four γc family cytokines to adoptively transferred T cell cultivation in vitro with various combination protocols will promote the expansion of T_SCM_ cells with enhanced capacities to engraft, persist and mediate prolonged immune attacks against tumor masses. In addition to acquiring T_SCM_ during the manufacturing phase ex vivo, they are expected to maintain and expand T_SCM_ after co-administration with autologous T cells. Nevertheless, the administration of these wild-type cytokines is associated with some obstacles such as non-specific toxicities, off-target effect, and inefficiency, and their engineered versions may make up for these deficiencies to a large extent. In this review, we outline the potential of the stemness of the transferred T cells and summarize the roles of wild-type IL-2, IL-7, IL-15 and IL-21 belonging to γc family cytokines in the production, maintenance, and expansion of T cells with stemness in ACT. We also introduce their engineered types prompting T_SCM_ induction and discuss the limitations and future directions of incorporating the four cytokines in stemness induction for T cell-based cancer immunotherapy.

## Potential of T cell stemness in ACT

**Fig. 1:**
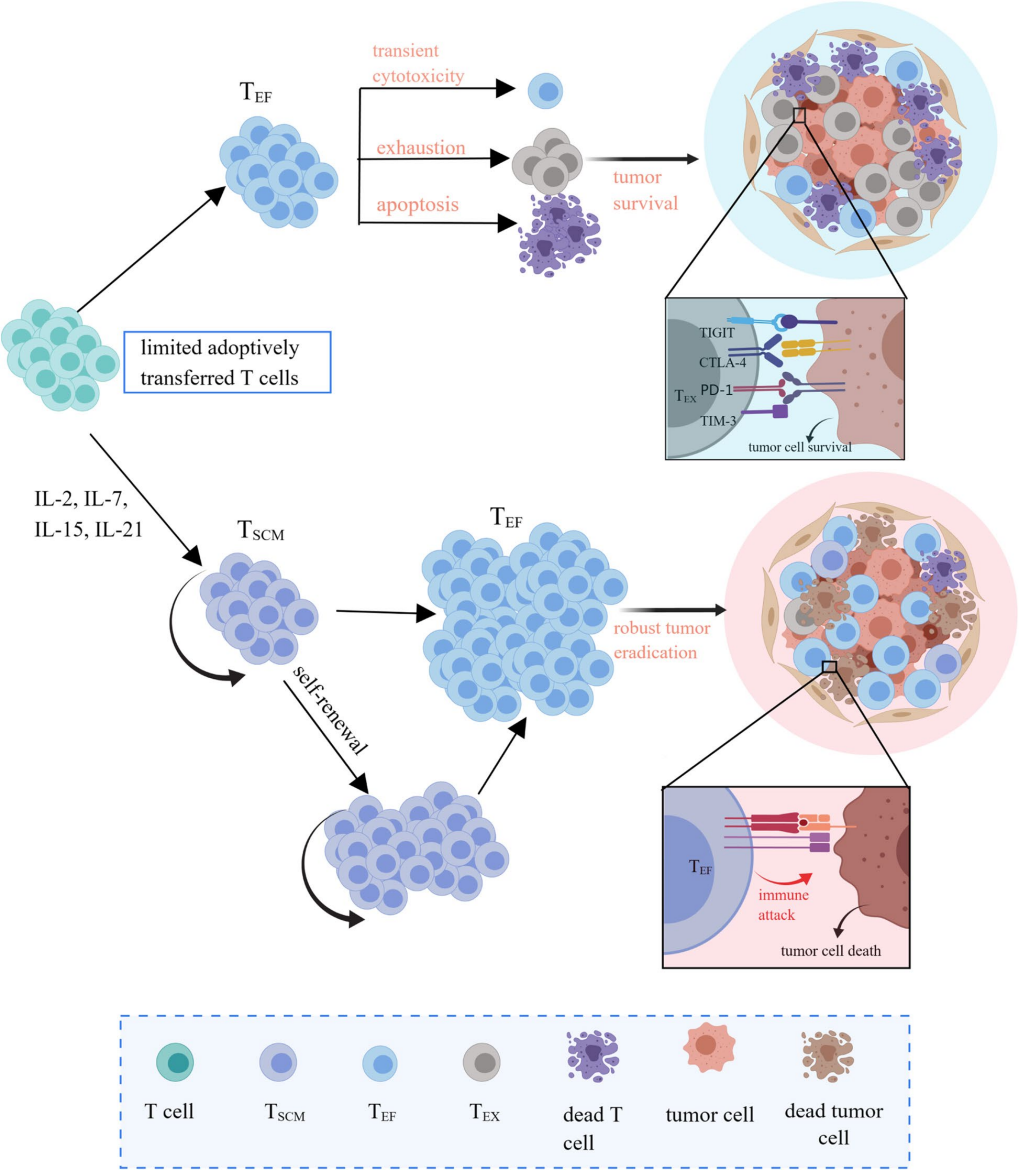
The potential of T_SCM_ for ACT. The limited adoptively transferred T cells inevitably experience apoptosis and exhaustion. Conventional infused effector T cells demonstrate transient cytotoxicity due to lack of complement, whereas infused T_SCM_ cells induced by the four γc family cytokines continually self-renew and produce enough effectors to mount a robust and persistent immune attack. ACT, adoptively cell therapy; T_EF_, effector T cells; T_SCM_, stem cell-like T cells; Tex, exhausted T cells

The efficacy of ACT largely depends on the status of adoptively transferred T cells. Low-differentiated T cells with stemness have elicited a significant superiority over conventionally activated T cells in tumor control, owing to the capability of enhanced self-renewal and persistence, as well as the rapid generation of effector subsets in vivo [34–36]. Regardless of the status, adoptive T_SCM_ cells and conventionally activated T cells without stemness are in the same suppressive tumor microenvironment (TME) and share a typical terminal response process, i.e., “transient cytotoxicity, consistent exhaustion, and ultimate apoptosis.” After infusion, adoptively transferred T cells are activated completely and dominantly differentiate into cytotoxic T lymphocytes (CTL), followed by the release of a bulk of cytotoxic molecules, such as perforin, granzyme B, and γ-interferon (γ-IFN), into the targeted synapse to achieve an ideal hit of tumor cell eradication. However, under an immunosuppressive microenvironment and continuous tumor antigen stimulation, most activated CD8(+) T cells upregulate the expression of co-inhibitory markers and exhibit an exhausted phenotype, culminating in a stepwise loss of cytotoxicity and non-response to immunotherapy [6, 13, 37] (Fig. 1). Beyond that, functional CTLs are subjected to activation-induced cell death (AICD) via high expression of the Fas/FasL axis, which further prevents an excessive immune response [16]. Remarkably, T_SCM_ cells with memory traits mediate a faster and stronger recall response on a lower threshold of antigen restimulation. Hence, a limited number of engineered T cells without stemness execute the limited tumor-killing effect and unavoidably go forward to exhaustion and apoptosis without sufficient supplements, whereas T_SCM_ cells preserve the ability of self-renewal and differentiation into better effector T cells to mount a robust antitumor response after substantial expansion (Fig. 1).

T_SCM_ cells have been elucidated to trigger complete tumor regression and durable response in hematopoietic malignancies and solid cancers [27, 34, 38–42]. In human B-cell malignancies, CD19-CAR-modified T_SCM_ cells present enhanced metabolic fitness and mediate long-lasting antitumor responses [39]. In patients with non-small cell lung cancer (NSCLC), T_SCM_ cells are located in peripheral blood, producing antitumor molecules. Relatively fewer T_SCM_ cells are found in lymph nodes, contributing to faster recall responses against cancer cells [43]. Therefore, conferring stemness to antitumor T cells might unleash the full potential of immunotherapies based on CD8(+) T cells. Disappointingly, terminally differentiated T cells are commonly enriched in the TME after ACT, and a lower T_SCM_ state of tumor-infiltrating T lymphocytes (TILs) exists in vivo [42]. Intra-tumor immune niches in which T_SCM_ cells reside are commonly deficient in patients with progressive tumors [44]. Thus, augmenting the pool of T_SCM_ cells, either by isolating and expanding intrinsic stem-like neoantigen-specific T cells or by engineering T cells to acquire stem-like attributes in vitro might provide promising opportunities for developing more effective T cell-based immunotherapies. Harnessing the generation of more T_SCM_ cells via γc family cytokines might lead to the development of potent cancer immunotherapy.

## Four cytokines of the γc family regulate T cell stemness

Cytokines containing the γc family serve as a communicative bridge among immune cells and non-immune cells in the TME, providing a crucial signal to regulate the ultimate differentiation of antigen-specific T cells and critically impact their cytotoxicity. The γc family of cytokines, including IL-2, IL-4, IL-9, IL-15, and IL-21, is a specific group of cytokines that share a common cytokine receptor γ chain. IL-4 is primarily recognized to promote humoral immunity and regulatory T cell (Treg) development, and IL-9 is thought to improve T_H_9 differentiation [45, 46]. In particular, it is four γc family cytokines, IL-2, IL-7, IL-15 and IL-21, that are crucial regulators of T cell-based cellular immunity and involved in orchestrating T cell stemness, contributing to enhanced antitumor activity in CAR-T therapy. Because of the great potential of T_SCM_ cells for tumor control, further understanding of how these cytokines orchestrate the induction of persistent T_SCM_ cells will contribute to the optimization of infused T_SCM_ cell production before transfer. Here, we respectively clarify the role of the four γc family cytokines in regulating the formation and expansion of T_SCM_ cells, as well as the underlying mechanisms.

### IL-2 contributes to terminal differentiation of CD8(+) T cells

**Fig. 2:**
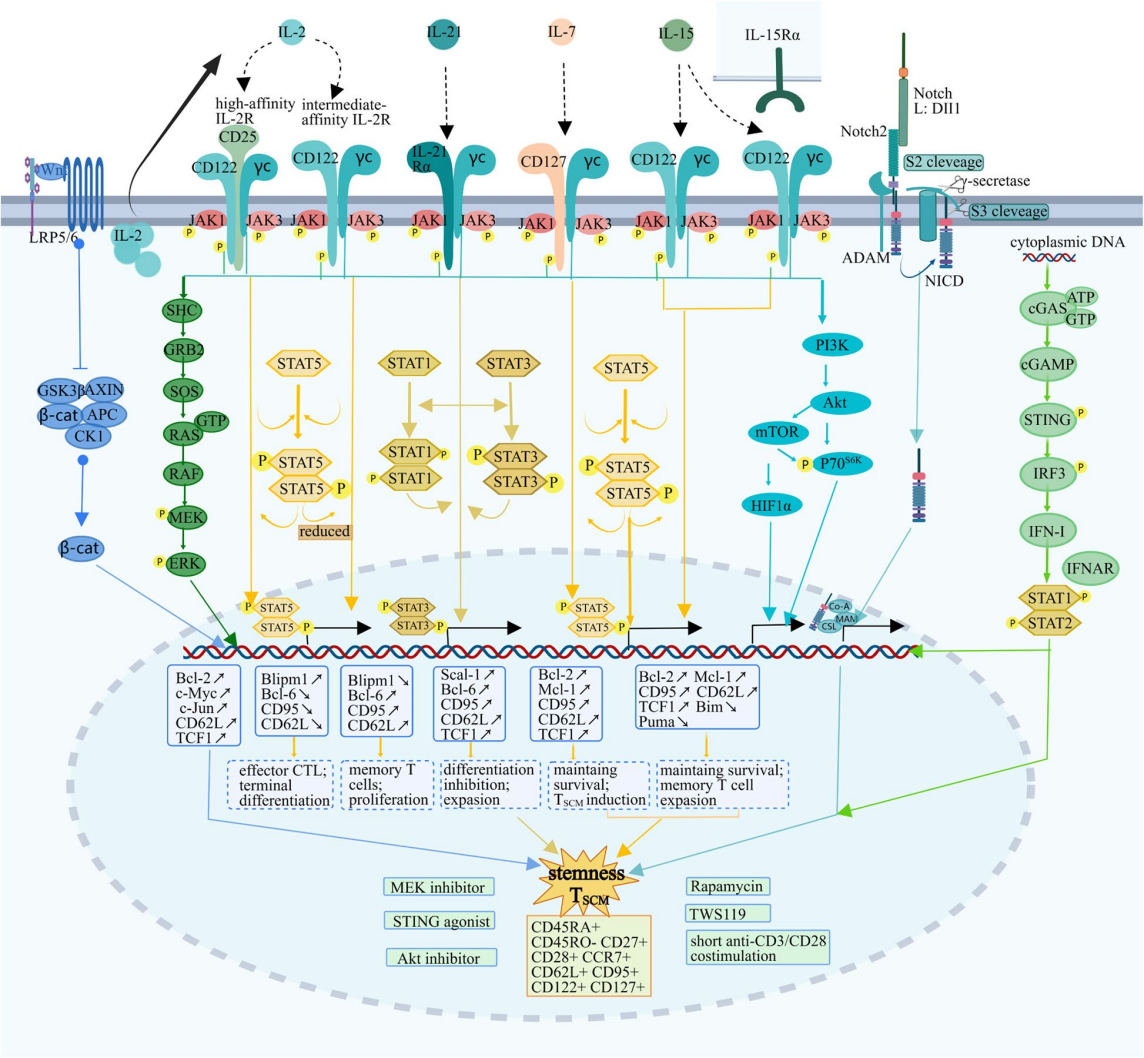
The signaling pathways mediated by the four γc family cytokines regulate T cell stemness. The stemness of T cells is cooperated by several signaling pathways. The four γc family cytokines trigger the JAK-STAT, RAS-MAPK, and PI3K-AKT signaling pathways to collectively modulate T_SCM_ phenotype induction. IL-2 combines with high-affinity IL-2R via dimeric STAT5 to induce terminal differentiation whereas reduced STAT5 signaling by intermediate-affinity IL-2R can increase the expression of memory- and antiapoptotic-associated molecules. IL-7 and the trans-presented IL-15 also activate STAT5 phosphorylation to initiate the expression of the stem-like markers such as CD95, TCF1, and CD62L, for persistent survival and T_SCM_ cell induction. IL-21 mainly activates the phosphorylation of STAT1 and STAT3, the latter of which induces the expression of Scal-1, CD95, TCF1 and CD62L, thereby contributing to T_SCM_ phenotype formation. The activation of P70^S6K^ via PI3K-AKT and mTOR is involved in T cell differentiation; therefore, inhibitors of the AKT pathway, such as AKT inhibitors and mTOR inhibitor Rapamycin, provide opportunities to regulate stemness. WNT inhibits GSK3β to release β-catenin into the nucleus to regulate the expressions of Bcl-2, c-Myc, c-Jun, CD62L and TCF1 to promote T_SCM_ formation, the same as the GSK3β inhibitor TWS119. In addition, the activation of the Notch and cGAS-STING pathways can also promote T cell stemness

IL-2 is discovered as a pleiotropic T cell growth factor [47, 48] mainly derived from CD4(+) T cells, and plays a major role in cellular immunity. Cellular immune responses are triggered by antigen encounter and TCR-CD3 activation and then amplified by the interaction of IL-2 and its receptors as the third signal. The high-affinity IL-2 receptor (IL-2R) comprised of α, β, and γc subunits is mainly distributed in activated effector T cells and Tregs. In contrast, memory CD8(+) T cells express intermediate-affinity heterodimeric IL-2R, which only includes IL-2Rβ/γc chains [49]. Regardless of affinity, both receptors can transmit signals through the recruitment of JAK1 and JAK3 by the intracellular domains of IL-2Rβ and IL-2Rγ respectively, as well as through the phosphorylation of tyrosine residues [50–52]. These transmitted signals can further activate several pathways in T cells, including the JAK1/3-STAT5, JAK-RAS-MAPK cascade, PI3K-mTORC1, and PI3K-AKT pathways [49, 52]. Among them, STAT5 signaling has been shown to promote the formation of terminal effectors [53–55] (Fig. 2).

With IL-2Rα (CD25) being upregulated by TCR-CD3 activation, the binding of IL-2 to high-affinity IL-2R enables CD8(+) T cells to become effector T cells and release cytotoxic molecules. To maintain immune homeostasis and prevent overactivation, some negative feedback molecules such as Fas/FasL and other inhibitory molecules gradually present on the effector T cells. Subsequently, they mediate T cell anergy and apoptosis at the advanced stage of the immune response, which is partially owing to the Treg cell-mediated immune suppression. With the constitutive expression of high-affinity IL-2Rα, Treg cells competitively deprive effector T cells of IL-2 to support their expansion and suppress the T cell response [56, 57]. A murine model with Treg cell-specific conditional knockout of high-affinity IL-2R was established, which showed that the deficiency of IL-2 consumption by Treg cells impaired their suppression of CD8(+) T cell proliferation [56], particularly that of memory T cells. In general, IL-2 combined with high-affinity IL-2R enhances the expansion of CD8(+) effector T cells and promotes their terminal differentiation both directly [57, 58] and indirectly by maintaining the suppressive function of Treg cells [52, 57, 59, 60]. In addition, IL-2 binds to the intermediate-affinity IL-2R to mediate a low level of IL-2 signaling for facilitating the expression of IL-7α and CD62 ligand (CD62L), and preferentially bring about memory T cells [52, 54]. These findings suggest that IL-2 has a dual and opposing function in regulating the CD8(+) T cell phenotype. High levels of IL-2 signaling drive CD8(+) T cells to differentiate into short-lived effector cells, while low levels of IL-2 promote the differentiation of long-lived memory T cells [52, 54]. An appropriate affinity of IL-2 for IL-2R may raise the possibility to develop and maintain a subset of memory T cells with persistent survival and self-renewal capacity, known as T_SCM_.

With this regard, by reducing IL-2 signal strength, CD8(+) T cells can be successfully cultivated in vitro to acquire stemness and mediate persistent tumor suppression in CAR-T therapy. Of note, IL-2-producing CD8(+) T cells demonstrated attenuated IL-2-dependent STAT5 signaling, probably resulting in the restriction of terminal differentiation. This finding was supported by the observation that a specific subset of CD8(+) T cells capable of synthesizing IL-2 during the effector phase attained stem-like memory traits and resisted exhaustion at the effector phase [55]. Similar to the lower signaling mediated by intrinsically generated IL-2, short-term culture with exogenous IL-2 promoted the CD62L^+^CCR7^+^ memory CAR-T cells possessing stronger propagating ability and better tumor control in vivo. In contrast, long-term culture drove terminal differentiation and dampened, rather than boosted the antitumoral function of CAR-T cells [61].

Beyond the lower dose and shorter incubation time of wild-type IL-2, engineered IL-2 and receptors may provide another feasible strategy for precise and efficient T_SCM_ induction. Wild-type IL-2 administration for cancer receives low complete response rates and poor tumor control due to its short half-life, which requires a very high amount of intravenous IL-2 associated with severe non-specific toxicities, and off-target effects on Treg cells [62–67]. Thus, some engineered IL-2 proteins with prolonged half-life are designed to improve cell targeting and selectivity for dimeric intermediate-affinity IL-2R, typically entailing the reduced interaction of IL-2 with the CD25 subunit or enhanced binding to CD122 [68–72]. They can be engineered by introducing mutations that shift the selectivity towards cells expressing intermediate-affinity IL-2R, yielding IL-2 muteins, orthogonal IL-2-IL-2R mutein pairs or fusion with other proteins including polyethylene glycol (Peg) (PEGylated IL-2), antibodies (IL-2 immune complexes), and the extracellular domain of CD25 (IL-2-CD25 fusion proteins) [71–73]. These engineered IL-2 proteins have the potential to preferentially target antigen-experienced memory T cells and NK cells that express dimeric intermediate-affinity IL-2R and manifest enhanced antitumoral responses in T cell-based therapy [74, 75], some of which partially benefit from the increased formation of T_SCM_. H9T, an engineered IL-2 partial agonist obtained via a single mutation Q126T in ‘superkine’ H9 that reduced the binding of H9 to IL-2Rγ, promoted the expansion of transferred CD8(+) T cells in vitro without terminal differentiation, and maintained a stem-cell-like state, which was attributed to reduced STAT5 signaling and increased T cell transcription factor 1 [76]. Intriguingly, much lower expression of exhaustion markers PD-1, TIM-3, and LAG-3 on infused T cells was induced by co-culturing with H9T in comparison with IL-2 or H9, which impaired the impediment to antitumor response and prolonged survival. As a result, the appropriately reduced binding of IL-2 to dimeric IL-2R may be a potential approach to promoting and maintaining the stem-cell-like phenotype of CD8(+) T cells without compromising the function of inducing amplification. To further reduce systemic toxicity due to IL-2 pluripotency in vivo, IL-2 and its receptor were engineered as an orthogonal cytokine-cytokine receptor pair, in which orthogonal IL-2 selectively interacts with its orthogonal receptor expressed on CAR-T cells capable of delivering an appropriate IL-2 signal in vivo [77]. IL-2 cytokine-receptor orthogonal pairs promote the specific expansion of orthogonal IL-2Rβ-modified T cells in vivo with negligible toxicity and improved antitumor response against leukemia and B16-F10 melanoma [77, 78]. Developed as orthogonal human IL-2, STK-009 selectively expanded orthogonal IL-2Rβ (hoRb)-expressing CAR-T cells and maintained the presence of T_SCM_in vivo, which delivered complete responses in refractory lymphomas [79]. Another orthogonal IL-2-IL-2R mutein pair, human chimeric orthogonal IL-2Rβ-ECD–IL-9R-ICD (O9R), fused orthogonal IL-2 receptor extracellular domain (ECD) with the intracellular domain (ICD) of IL-9R such that the orthogonal IL-2 elicited the corresponding γc cytokine signal [10]. Mediating a reduced STAT5 signal compared to O2R, orthogonal IL-2 drove stemness and superior effector capacity in O9R-expressing TCR-or CAR-T cells in mouse solid tumor models of melanoma and pancreatic cancer. Furthermore, compared with the direct co-administration of wild-type or engineered IL-2 in vivo with T cell transfer, synthetic cytokine circuits such as tumor-specific synNotch receptors and synthetic zinc finger transcription regulators (synZiFTRs) on engineered T cells allowed the precise production of IL-2 in time and space to achieve less systemic toxicity [74, 80]. In contrast to the lower dose and shorter incubation time of IL-2 during the manufacturing phase in vitro, the suitable alteration of IL-2 and IL-2R in an engineered manner may inspire a more efficient way to not only precisely improve the induction of targeted T cell stemness in vivo but also alleviate the side effects caused by its pleiotropy, which gives rise to adoptively transferred T_SCM_ cells and mediates continual responses in ACT.

### IL-7 induces T_SCM_ cell differentiation and long-term longevity

Unlike IL-2, IL-7 was first identified as a stromal cell-derived factor and was encoded from human cDNA in vitro [81, 82]. It signals through IL-7R containing IL-7Rα (CD127) and γc subunits with activation of the JAK-STAT and PI3K-AKT pathways. Intriguingly, IL-7 is essential for T cell development and for maintaining and restoring CD8(+) memory T cell homeostasis alone or together with IL-15, another γc family cytokine illuminated later [83]. During thymopoiesis, IL-7R is present on double-negative (DN) T cells, absent on double-positive (DP) T cells, restored on single-positive (SP) T cells, and retained on mature T cells in the thymus [84], indicating that T cell development is closely related to the controlled expression of IL-7R. During mature T cell differentiation in peripheral lymphoid organs, in contrast to other γc family cytokines, IL-7R is highly expressed on naïve T cells but lost on the most effector T cells after TCR activation, and then re-expressed on memory T cells. Exceptionally, a small minority of effector T cells with increased IL-7R expression are predisposed to differentiation into memory cell subsets that persist for a long time in vivo, implicating that IL-7/IL-7R serves as a critical regulator of memory T cell transition and maintenance.

The mechanism underlying IL-7-mediated regulation of the survival of long-lived memory T cells is explicitly associated with the reprogramming of energy metabolism to some extent, including lipid metabolism and oxidative phosphorylation. The glycerol channel aquaporin 9 (AQP9)-dependent triglyceride (TAG) synthesis driven by IL-7 is indispensable for promoting the longevity of memory CD8(+) T cells [85]. Likewise, IL-7 increases glucose uptake by T_SCM_ cells via overexpression GLUT1 and upregulation of the glycolytic enzyme hexokinase 2 (HK2), as illustrated by the inhibition of T_SCM_ cell generation and expansion using the selective glucose uptake inhibitor WZB117 [86, 87]. Additionally, sustained expression of the antiapoptotic proteins BCL-2 and Mcl-1 in response to IL-7 is also involved in the survival of memory T cells [88–90] (Fig. 2). Moreover, IL-7 is controlled by a negative regulatory feedback loop to maintain homeostasis of memory T cells [91, 92]. Therefore, IL-7 highlights the considerable potential for an efficient transition to long-lived T_SCM_ cells and enhanced antitumor responses by elevating their expansion.

### IL-15 promotes T_SCM_ cell phenotype expansion in vitro

IL-15 is commonly produced by a wide range of cells and acts on various immune cells like T cells through IL-15 receptors (IL-15R) to serve as a T cell growth factor [93, 94]. IL-15R is comprised of three subunits: IL-15Rα (CD215), IL-15/IL-2Rβ (CD122), and γc (CD132). Since two members of the γc family cytokines, IL-2 and IL-15, share IL-2Rβ, they consequently share some common biological properties, which was proven by evidence that IL-2Rβ deficiency impeded T cell proliferation induced by IL-15 [93]. By contrast with IL-2, which induces effector T cell terminal differentiation through high-affinity IL-2R, IL-15 remarkably tends to promote the maintenance and expansion of memory CD8(+) T cells owing to the unique IL-15Rα [95–98]. More strikingly, the distinct subunit IL-15Rα mainly presents IL-15 in trans to neighboring cells, including memory T cells, by antigen-presenting cells (APCs) [99]. As an autonomous and antigen-independent process [99], the trans-presentation of IL-15 provides sufficient signals to sustain antigen-specific memory CD8(+) T cell survival and expansion in the absence of antigens [95, 100–103]. The underlying mechanism is that membrane IL-15Rα on APCs captures IL-15 with high affinity and trans-presents IL-15 to activate the IL-2/15Rβγ heterodimer of memory CD8(+) T cells, subsequently activating the same JAK-STAT pathway as IL-2/IL-2R [104]. The phosphorylated STAT5 proteins form heterodimers to regulate the expression of downstream target genes, involving the upregulation of the antiapoptotic protein Bcl-2 and NF-kB signaling and the downregulation of the expression of pro-apoptotic molecules Bim and Puma [105, 106]. Beyond the JAK-STAT pathway, IL-15-induced proliferation of memory CD8(+) T cells partially relies on activation of the RAS-RAF-MAPK cascade and PI3K signalings [95, 106]. Besides, the increased activity of mTORC1 [101] and FKBP12 (FK506-binding protein 1 A, also known as FKBP1A) driven by IL-15 activates P70 S6 kinase and is responsible for promoting the cell cycle progression of memory T cells [95, 100] (Fig. 2).

As IL-15 mediates a lower mTORC1 activity than IL-2 to prevent T cell terminal differentiation, co-culture with IL-15 alone is superior to that with IL-2 alone in preserving the T_SCM_ phenotype during the expansion process of CAR-T cells ex vivo [107]. Upon tumor challenge, CAR-T cells exposed to IL-15 exhibited fewer apoptotic features, higher proliferative capacity, and a superior antitumor response than those exposed to IL-2 in vivo. In addition, memory CD8(+) T cells were found to expand significantly following 3-day administration of recombinant human IL-15 (rhIL-15), among which T_SCM_ cells were also observed a profound tendency to expand in phase I clinical trial [108]. However, its short half-life and low availability limit its application in vivo. Thus, different IL-15 derivatives by fusion with soluble IL-15Rα, Fc domain, or Peg have been engineered for stable bioactivity to overcome these barriers [109–113]. To further prolong the persistence of infused T cells, stable IL-15/IL-15Rα complexes were incorporated to induce and sustain the expansion of the CD62L^+^ and CCR7^+^ central memory T cell (Tcm) phenotype ex vivo resulting in augmented efficacy of adoptive immunotherapy [114]. As a result, efforts have been made to optimize the IL-15/IL-15Rα structure to achieve a higher proportion of infused T cells with stemness and greater antitumor potency in ACT [115]. In addition to its incorporation in ex vivo precultures, IL-15 and its receptor complex have been integrated into CAR engineering to maintain T_SCM_ expansion in vivo for durable responses [116–122]. To mimic the trans-presentation of IL-15 in the context of IL-15Rα, membrane-bound chimeric IL-15 (mbIL15) was generated by the fusion of native IL-15 to IL-15Rα via a flexible linker. It was co-expressed with second-generation CAR, yielding mbIL15-CAR-T cells that retained long-term persistence and memory potential with a T_SCM_-like phenotype [116]. Furthermore, to attenuate IL-15-induced off-target toxicity, a next-generation tumor-conditional IL-15, called pro-IL15, was developed to fulfill tumor-targeted delivery by fusing the extracellular domain of IL-15Rβ into the N-terminus of IL-15-IL-15Rα-Fc (super IL-15-Fc) using a peptide linker specifically cleaved by matrix metalloproteinase (MMP) inside the TME [123]. In mouse tumor models, pro-IL-15 significantly increased the proportion of stem-like CD8(+) T cells in tumor tissue and enhanced sensitivity to immune checkpoint inhibitors [123].

### IL-21 drives the development of naïve T cells into T_SCM_ cells

IL-21 is mainly derived from activated CD4(+) T cells and NK cells and demonstrates broad pleiotropic effects on the immune system [83, 124, 125]. Similar to IL-2, the binding of IL-21 to a functional IL-21 receptor (IL-21R) consisting of heterodimers of γc (CD132) and specific chain IL-21Rα (CD360) stimulates the phosphorylation of tyrosine residues to activate several downstream signals covering the JAK-STAT1/3, PI3K-AKT and MAPK signaling pathways (Fig. 2). Among these, STAT3 phosphorylation is involved in IL-21-induced T_SCM_ cell formation by upregulating the expression of the memory-associated transcriptional factors notch, TBX21, and SOCS1, and downregulation of mature effector markers Eomesodermin (EOMES) and GATA Binding Protein 3 (GATA3) [38]. Besides, metabolic reprogramming by IL-21 is also responsible for orchestrating memory CD8(+) T cell differentiation with stemness. When cultured with IL-21 in vitro, CD8(+) T cells elicit a metabolic skewing away from aerobic glycolysis towards a naïve-like metabolically quiescent state characterized by oxidative phosphorylation and fatty acid oxidation (FAO) with increased mitochondrial fitness and biogenesis [126, 127].

Therefore, IL-21 modulates the differentiation of memory CD8(+) T cell subsets as a critical threshold for the generation of memory stem-like CD8(+) T cells from naïve T cells [38, 39, 128–130]. IL-21 alone preferentially impedes terminal differentiation and improves memory subset formation of T cells [38, 128]. In the exploration of the conditions used to raise T_SCM_ cellsfrom naïve T cells ex vivo, the addition of IL-21 helped transferred T_SCM_ cells maintain their differentiation stage and potential for an increased response after short-time anti-CD3/CD28 co-stimulation in adoptive immunotherapy [129]. When synergized with other factors, IL-21 exerts a pivotal role in T_SCM_ cell attainment and expansion, exemplified by the synergy with lactate dehydrogenase (LDH) inhibitor in a mouse model of pmel-1 specific TCR-T cell adoptive cancer immunotherapy [127]. When treated with a combination of LDH inhibitor and IL-21 in vitro, naïve pmel-1 CD8(+) T cells showed a naïve-like metabolic immunophenotype similar to that with IL-21 alone, which promoted the production of CD44^low^ CD62^high^ Sca1^high^ cells and induced the suppression of exhaustion markers LAG3, PD1, 2B4, and TIM3, typically of T_SCM_ cells [127]. Furthermore, IL-21 augmented rapamycin in the maintenance and expansion of AFP peptide-specific T_SCM_ cells in vitro [39] and induced the CD19-CAR-modified T_SCM_ cells from naïve precursors with the GSK-3β inhibitor TWS119 and IL-7, which showed superior elimination of tumors [39]. Strikingly, PD-1Ab21, a fusion protein of anti-PD-1 antibody and IL-21, which was successfully developed to block the interaction of PD-1 on T cells with PD-L1 and targeted IL-21 on PD-1^+^ T cells simultaneously, is expected to further stimulate the differentiation of activated T cells back to T_SCM_ cells mediated by the IL-21 receptor in vitro [130]. In tumor-bearing mice, stronger tumor remission was observed with PD-1Ab21 treatment than that with the combination of PD-1 blockade and IL-21 infusion, which was attributed to the increased frequency of T_SCM_ cells and robust expansion of tumor-specific CD8(+) T cells with a memory phenotype. In summary, IL-21 plays a crucial role in the induction and maintenance of transferred T_SCM_ cells with enhanced antitumor and self-renewal capacities, which has significant implications for adoptive T cell-based immunotherapy.

### Different combinations of the four cytokines for an optimal protocol to acquire efficient T_SCM_ cells

Taken together, IL-2, IL-7, IL-15 and IL-21 are jointly involved in T cell differentiation and play different roles in T cell stemness. IL-2 drives T cell activation and terminal differentiation, while IL-21 prompts naïve T cells to T_SCM_ cells phenotype. Meanwhile, IL-7 enables T_SCM_ cells to form and maintain and IL-15 primarily stimulates their robust expansion [131]. Their different functions imply a multi-target strategy that different combinations might be an appropriate way to manufacture T_SCM_ cells in vitro for ACT. From naïve precursor CD8(+) T cells, T_SCM_ cells were generated by culturing with IL-7 and IL-15 in vitro [132, 133] or in the presence of IL-7/IL-21 and GSK-3β inhibitor TWS119 [39]. Except for promoting the generation of T_SCM_ cells, adding reduced TCR stimulation to IL-7/IL-15 prevented terminal differentiation to efficiently maintain the stemness phenotype for a long time [134]. Moreover, effector T cells were demonstrated to convert into T_SCM_-like cells by IL-7 and IL-15 [23]. In a preclinical model, autologous reoriented CD19-CAR-T cells were incubated with IL-7 and IL-15 to obtain and preserve the T_SCM_ cell subpopulation, which was transferred into patients with B-cell malignancies and produced stronger antitumor responses [135]. In many clinical trials, IL-7 combined with IL-15 has been used to induce adoptively transferred T cell stemness in order to prolong survival in vivo and mediate continual responses against tumors in adoptive cell immunotherapy (Table 1). As mentioned above, the transferred T_SCM_ subset was efficiently induced and maintained by the combined utilization of IL-15 and IL-7, showing a better antitumoral effect for adoptive T-cell therapy [133, 135]. To characterize an applicable and efficient combination of different cytokines, Gargett et al. showed that it was more accessible to the acquisition of the T_SCM_ phenotype by co-culture with IL-7/IL-15 than that with IL-2/IL-21 [136]. Nevertheless, Alizadeh et al. [107] demonstrated that inclusion of IL-7 and/or IL-21 impaired the effect of IL-15 on stem-like phenotype maintenance and antitumor potency [107]. Therefore, the optimal protocol of the four γc family cytokines, which produces effective infused T_SCM_in vitro to mediate a robust clinical outcome for ACT remains opaque and needs further investigation. Interestingly, targeted inducible delivery of the four crucial cytokines into the tumor focus may be another strategy for T cells to directly induce intrinsic stemness formation in vivo and reprogram the TME [137, 138].

**Table 1:** γc family cytokines in clinical trials about ACT

Cytokines	NCT Number	Phase	Status	Tumor Type	Treatment of cytokines	ACT Type	The Role of Cytokines for T cells	Year
IL-2	NCT03475134	I	Active, not recruiting	Metastatic melanoma	High dose IL-2 after ACT	TIL	Promoting activation and proliferation	2018
	NCT03171220 [139]	I/II	Unknown status	Advanced malignant solid tumor	IL-2 after ACT; NRTs combined with PD-1 inhibitor (SHR-1210)	Neoantigen reactive T cells (NRTs)	Promoting immune response after ACT	2017
	NCT01659151	II	Active, not recruiting	Metastatic melanoma	High dose IL-2 after ACT	TIL	Promoting immune response after ACT	2012
	NCT02278887 [140]	III	Active, not recruiting	Metastatic Melanoma	High dose bolus IL-2 after infusion	TIL	Keeping the TIL active after ACT	2014
	NCT05505812	Early phase I	Not yet recruiting	Advanced breast cancer	IL-2 after ACT	autologous TIL infusion (HS-IT101)	Promoting immune response after ACT	2022
	NCT05475847	I	Recruiting	Cervical cancer	IL-2 after ACT	Autologous TIL(C-TIL052A)	Promoting immune response after ACT	2022
	NCT05361174	I/II	Recruiting	Unresectable melanoma, Metastatic melanoma, Stage III non-small cell lung cancer, Stage IV non-small cell lung cancer	IL-2 after ACT	Genetically modified autologous TIL(IOV-4001)	Promoting immune response after ACT	2022
	NCT05194735	I/II	Recruiting	Gynecologic cancer, Colorectal cancer, Pancreatic cancer, NSCLC, CHOL, OV, Endometrial Cancer, Ovarian carcinoma Ovary neoplasm, Squamous cell lung cancer, Adenocarcinoma of lung, Adenosquamous cell lung cancer	Aldesleukin (IL-2) with TCR-T treatment	Neoantigen specific TCR-T cell	Supporting growth and activation of TCR-T cells	2022
	NCT05141474	Early phase I	Recruiting	Epithelial Tumors, Malignant, Malignant Solid Tumor	IL-2 infusion after ACT	Next-generation Neoantigen-selected TIL (NEXTGENTIL-ACT)	supporting the expansion of the infused cells.	2021
IL-7	NCT04833504	Clinical follow-up study	Completed	Diffuse large B-cell lymphoma, Mantle cell lymphoma, Transformed follicular lymphoma, Primary mediastinal large B-cell lymphoma	CD19-CAR-T expressing IL-7	CD19-CAR-T expressing IL-7 and CCL19(CD19-7 × 19 CAR-T treatment)	helping CAR T cells grow better and stay in the blood longer	2021
	NCT04381741	I	Recruiting	Diffuse large B-cell lymphoma	CD19 CAR-T expressing IL-7	CD19-7 × 19 CAR-T plus PD1 monoclonal antibody	Promoting the survival of CAR-T cells in lymphoma tissue	2020
	NCT03932565	I	Recruiting	Nectin4-positive advanced malignant solid tumor	expressing IL-7 of CAR-T cells	Nectin4/FAP-targeted fourth-generation CAR-T cells (expressing IL-7 and CCL19, or IL-12)	Promoting survival	2019
	NCT03929107	II	Recruiting	B cell lymphoma	expressing IL-7 of CD19-CAR-T cells	Interleukin-7 and chemokine (C-C motif) ligand 19-expressing CD19-CAR-T cells	Promoting the survival	2019
	NCT03198546 [141]	II	Recruiting	Hepatocellular carcinoma	With IL-7/CCL19 secreting vector	GPC3/TGFβ-CART cells	Promoting the survival and expansion	2017
C7R(IL-7R)	NCT04099797	I	Recruiting	Diffuse intrinsic pontine glioma, High grade glioma, Embryonal tumor, Ependymal tumor	Expressing constitutively active IL-7 receptors	Autologous T lymphocytes expressing GD2-specific chimeric antigen and constitutively active IL-7 receptors (C7R-GD2.CART cells)	Giving the cells a constant supply of cytokine and helping them to survive for a longer period	2019
	NCT03635632	I	Recruiting	Relapsed or refractory neuroblastoma and other GD2 positive cancers (GAIL-N)	Expressing constitutively active IL-7 receptors	C7R-GD2.CART cells	Giving the cells a constant supply of cytokine and helping them to survive for a longer period	2018
IL-15	NCT05103631	I	Recruiting	Liver Cell Carcinoma	Engineered CAR-T with IL-15	Interleukin-15 armored glypican-3-specific chimeric antigen (GPC3-CAR) receptor expressing autologous T Cells (CATCH T cells)	helping CAR T cells grow better and stay in the blood longer	2021
	NCT04377932	I	Recruiting	Pediatric solid tumors: Liver cancer, Rhabdomyosarcoma, Malignant rhabdoid tumor, Liposarcoma, Wilms tumor, Yolk sac tumor	Engineered CAR-T with IL-15	Interleukin-15 armored GPC3-CAR expressed in T cells (AGAR T cells)	helping CAR T cells grow better and stay in the blood longer	2020
	NCT03721068	I	Recruiting	Neuroblastoma Osteosarcoma	Engineered CAR-T with IL-15	iC9.GD2.CAR.IL-15 T-cells, GD2-CAR-T cells expressing IL–15, and the inducible caspase 9 safety switch (iC9)	allowing the CAR-T cells to survive and grow in vivo	2018
	NCT04844086	I	Terminated	Advanced Lymphoid Malignancies	Engineered CAR-T with mbIL-15	RPM CD19-mbIL15-CAR-T cells	allowing the CAR-T cells to survive and grow in vivo	2021
IL-7 + IL-15	NCT04186520 [142]	I/II	Recruiting	Relapsed refractory B cell malignancies	IL-7/IL-15 pre-treating CAR-T cells with flexible 8/12-day manufacturing and a fixed 12-day manufacturing process	CAR-20/19-T cells	Promoting CAR-T cells survival and expansion	2019
IL-2 vs. IL-7/IL-15	NCT02992834	IV	Unknown status	CD19^+^ B cell lymphoma	IL-2 vs. IL-7/IL-15 pre-treated CD19 TCR-T cells	Anti-CD19: TCRζ Chimeric Antigen Receptor-T Cells	Promoting expansion and survival	2016
	NCT02652910	I/II	Unknown status	B cell lymphoma	Manufacturing CD19.CAR-T cells through IL-7/IL-15 or IL-2-mediated expansion	anti-CD19 CAR-T cells	IL-2: generating terminally differentiated effector cells; IL-7/IL-15: helping to selectively expand CAR-T cells with various memory phenotypes and improve persistency	2016
IL-7 + IL-21	NCT01087294 [143]	I	Active, not recruiting	Recurrent or persistent B-cell malignancies	Culturing CAR-T cells in media containing IL-21, IL-7, and TWS119	Anti-CD19-CAR T cells	Helping differentiation into long-lived T cells and improving survival	2010
IL-2; IL-15 + IL-21;	NCT04729543	I/II	Recruiting	Melanoma; Melanoma, uveal; Head and neck cancer	IL-15 and IL-21 cultivation; low dose of IL-2 administrations after ACT	autologous MC2 TCR T cells	Using IL-15 and IL-21 to generate young T cells; supporting T cells response by IL-2	2021
IL-15 + IL-21	NCT04093648	I	Withdrawn	Hepatocellular Carcinoma Hepatoblastoma	Engineered CAR-T with IL-15 plus IL-21	Interleukin-15 and − 21 armored glypican-3-specific chimeric antigen receptors expressed in T cells (CARE T cells)	helping CAR T cells grow better and stay in the blood longer	2019
	NCT04715191	I	Not yet recruiting	Liver cancer, Rhabdomyosarcoma, Malignant rhabdoid tumor, Liposarcoma, Wilms tumor, Yolk sac tumor	Engineered CAR-T with IL-15 plus IL-21	Interleukin-15 and − 21 armored glypican-3-specific chimeric antigen receptors expressed in T cells (CARE T cells)	helping CAR T cells grow better and stay in the blood longer	2021

## Other cytokines and signalings that regulate T cell fate and promote T_SCM._

In addition to the four γc family cytokines, there are other cytokines related to regulating T-cell fate, such as proinflammatory IL-1β, IL-18 and anti-inflammatory transfer growth factor β (TGF-β). A study [144] demonstrated that as effective proinflammatory cytokines, the increased production of IL-1β and IL-18 upon TIM-3 loss by dendritic cells (DCs) facilitated the maintenance of stem-like cells. To further identify the limited efficacy of recombinant IL-18 in clinical trials, an engineered “decoy resistant” IL-18 (DR-18) was designed to be impervious to IL-18BP inhibition [145], a high-affinity IL-18 receptor upregulated in various tumors and impedes the antitumor activity of IL-18. As a result, DR-18 not only maintained signaling potential but also exerted a robust antitumor activity by expanding the pool of stem-like TCF1^+^ precursor CD8(+) memory T cells and decreasing T cell exhaustion [145]. In addition, pre-stimulation with the combination of IL-12 and IL-18 contributes to memory T cells proliferation [146], and engineering T cells with scIL-12 and DR-18 demonstrates potent antitumoral effects [147]. Subsequently, membrane-bound form of IL-12 (mbIL12) engineered T cells were designed to improves potency of CAR-T cells both *in vitro and in vivo* [148]. Served as a typical anti-inflammatory cytokine, TGF-β is accepted for suppressing T cell activation and expansion [149–151]. As expected, TGF-β prominently impaired IL-7-induced memory T cell proliferation including T_SCM_ cells [150]. Inhibition of TGF-β signaling by either a TGF-β antibody or a small molecule inhibitor augmented the generation of CD62^high^CD44^high^ central memory CD8(+) T cells effectively [151]. However, exposure to TGF-β ex vivo resulted in the augmentation of early memory T cells through the downregulated expression of Blimp-1 and upregulated of the memory-associated transcription factor ID3 [149].

Apart from cytokines, several signaling pathways in T cells play a crucial role in the formation of the T_SCM_ phenotype, including the Notch, Wnt-β-catenin, mTOR, and cGAS-STING signaling pathways, as well as c-Myb (Fig. 2). A long time has witnessed that Notch signaling could influence the lineage commitment of T cells as well as maintain the memory T cell survival [152, 153]. Expressing a Notch ligand, Delta-like 1 (OP9-hDLL1), stromal OP9 cells were used to generate T_SCM_-like cells in vitro from activated T cells successfully and converted conventional human CAR-T cells into T_SCM_-like CAR-T cells through Notch-FOXM1 axis [22–24]. Similarly, the utilization of Wnt-β-catenin signaling suppression, which includes inhibitors of GSK-3β or the Wnt protein family member Wnt3α, arrested T cell differentiation into terminal effector cells and promoted the generation of self-renewing multipotent CD8(+) memory stem cells characterized by CD44^low^CD62L^high^Sca-1^high^CD122^high^Bcl-2^high^ [25–27]. Additionally, Stoycheva et al. discovered that deficiency of IFN-γR signaling promoted the formation of long-lived memory CD8(+) T cells and their sensitivity to weak TCR stimulation, which was correlated with reduced activation of mTOR and the accumulation of long-lived CD62L^high^Bcl-2^high^Eomes^high^ stem-like memory T cell precursors [154]. Simultaneously, the inhibition of mTORC1 in human naïve T cells after stimulation contributes to the induction of T_SCM_ cells [28]. In addition, the cGAS-STING mediated DNA sensing pathway in T cells is essential for antitumor immune responses and promotes the maintenance of stem cell-like CD8(+) T cells mechanistically by regulating transcription factor TCF1 expression [29]. Another factor involved in T cell stemness is the transcription factor c-Myb. It can promote stemness by inducing pro-memory and survival programs via TCF7 and Bcl2 and restricting the terminal differentiation [30]. Collectively, multiple signaling pathways in T cells work together to regulate the fate of T cell differentiation and play a key role in antitumor immunity.

## Combination of stemness promotion and exhaustion inhibition of T cells achieves further tumor eradication

Leveraging γc cytokines to induce T cell stemness contributes to a stable T_SCM_ pool to give rise to sufficient quantities of T cells for sustained immune elimination, partially alleviating the poor persistence of T cells in the TME. However, T cells stemmed from T_SCM_ will inevitably become dysfunctional and exhausted when continuously exposed to antigens in the TME. Therefore, integrated efforts of preserving T_SCM_ pool using γc cytokines and reinvigorating exhausted T cell are promising strategies to enhance antitumor immunity.

**Fig. 3:**
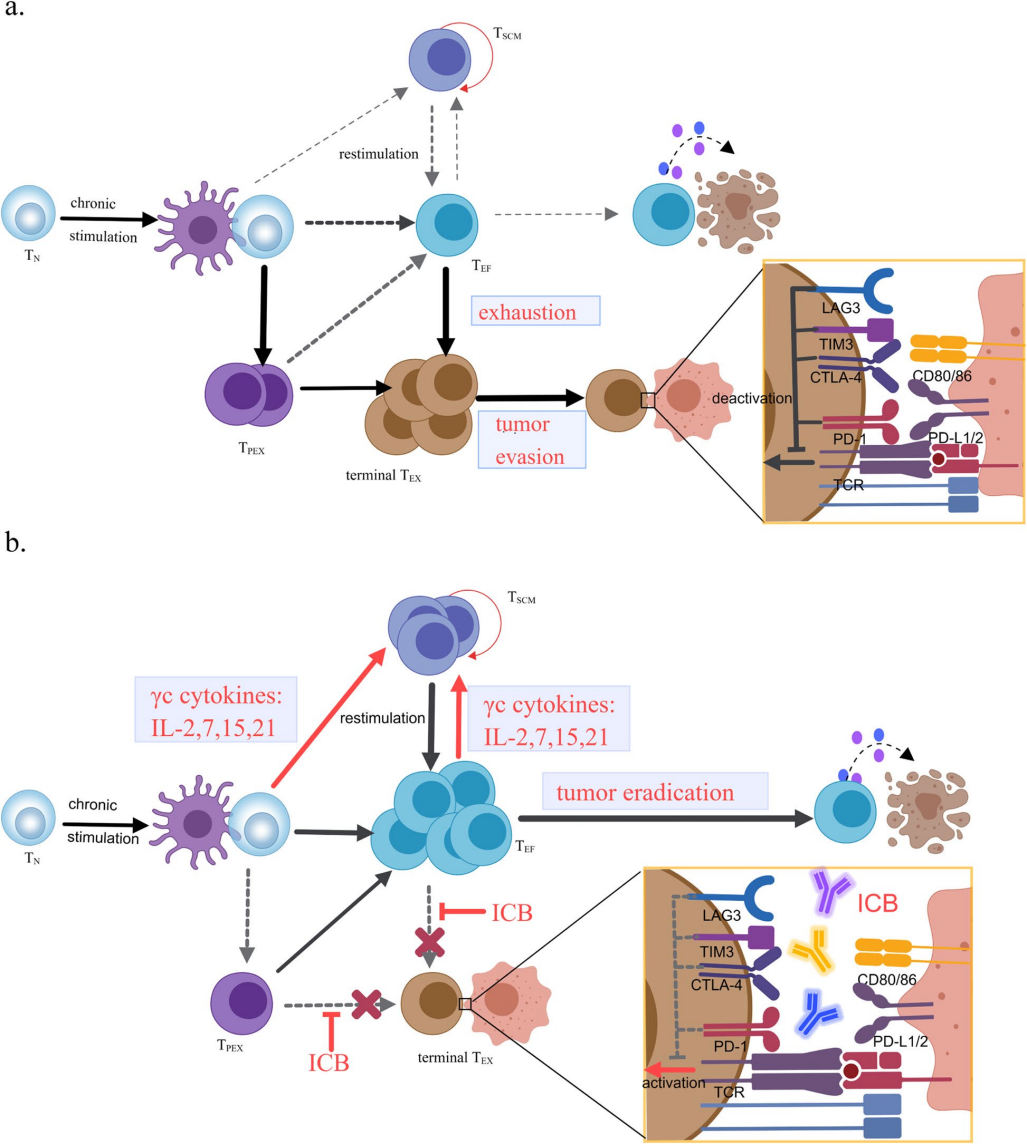
The combination of T_SCM_ formation by the γc family cytokines and ICB.** A** irreversibly terminal T_EX_ formation and exhaustion in the TME. **B** the combination of the γc cytokines IL-2, 7, 15, 21 and ICB efficiently promotes abundance of T_EF_ for tumor eradication

Exhaustion is cell adaptation of T cells in response to chronic antigen stimulation in chronic viral infection and tumors [155–159], with the aim of maintaining moderate levels of inflammatory responses while obviating excessive tissue damage. Exhausted T cells (T_EX_) are heterogenous and comprised of progenitor exhausted T cells (T_PEX_), intermediate T_EX_, and irreversible terminally differentiated T_EX_ [158, 160]. T_EX_ are phenotypically different from memory/effector T cells and hallmarked by upregulation of multiple inhibitory receptors, altered transcriptional and epigenetic profiles, and progressive loss of effector functions, capabilities of proliferation, and cytokine secretions [156, 158, 161–164] (Fig. 3). Proinflammatory cytokines featured by IL-2, interferon-γ (IFN-γ), and tumor necrosis factor-α (TNF-α) are fundamentally important for the survival, proliferation, and cytotoxicity of T cells. Particularly, IL-2 production in the TME is required for T cells to proliferate and elicit potent antitumor immune responses, while impaired secretion of IL-2 greatly dampens T cell activity [165, 166]. Besides the deficiencies in cytokine release, T_EX_ harbors increased expression of inhibitory receptors including PD-1, CTLA-4, LAG-3, TIM-3, TIGIT, CD160, and 2B4, which play pivotal roles in modulating the length and magnitude of immune responses and avoiding unrestrained cytotoxicity of T cells. Tumor cells excel in taking advantages of elevated inhibitory ligand/receptor axis to enable immune escape [155, 157, 159, 167, 168]. Activation of inhibitory ligand/receptor cascade signaling not only competes with target receptors or ligands that mediate activation signaling [169], but also attenuates signals from activated receptors via intracellular regulations [170]. Furthermore, the patterns of co-expression of inhibitory receptors and the quantities of inhibitory receptors on T cells substantially determine the intensity of T cell exhaustion [155, 169, 171–173].

The advent of T cell exhaustion is a major obstacle to complete elimination of target cells in adoptive cell therapy (ACT) [157]. Immune checkpoint blockade (ICB) targeting inhibitory receptors and their ligands have revolutionized antitumor therapies by “releasing the brakes” and potentiating T cell activities [165, 168, 174, 175]. Blockade of PD-1/PD-L1 interactions restored T cell functions and demonstrated impressive efficacy in controlling tumor outgrowth [176, 177], which could be attributable to reversed T cell exhaustion. Recent studies have revealed that PD-1/PD-L1 blockade preferentially expands “stem-like” T_PEX_ with self-renewal capacity and “effector-like” transitioning intermediate T_EX_ rather than irreversible terminally differentiated T_EX_ [170, 178–181]. Furthermore, combined targeting of PD-1 and CTLA-4 displayed better efficacy than the monotherapy [182–185], though the combination caused concerns of increased toxicity. More inhibitory receptors, such as TIM-3, LAG-3, and TIGIT, have gradually been leveraged as targets for therapeutic intervention [173, 186–189]. IL-2 replenishment has also been explored to alleviate T cell exhaustion ex vivo [165], and IL-2 replenishment plus PD-1/PD-L1 blockade has enriched better-functioning T cells in vivo and reprogrammed T cell exhaustion [190] via the orchestration that IL-2 stimulates antigen-specific T cells with stemness to expand and differentiate into effector cells and PD-1/PD-L1 blockade mitigates exhaustion and improves the antitumor capacity.

The elegant combination of IL-2 replenishment and PD-1/PD-L1 blockade raises the hypothesis that while “stepping on the accelerator” of immunity by increasing the sources of T cells, exhaustion should be mitigated to “release the brake”. Thus, it is both the quantities and quality of functional T cells that contribute to superior response in immunotherapy. The four γc family cytokines, IL-2, IL-7, IL-15, and IL-21, co-operate to form T_SCM_ pool, thereby improving T cell reserve. ICB are well-established agents that polarize T cells to more cytotoxic phenotype. Collectively, the synergy of γc family cytokines with ICB might be a paradigm-shifting combination for immunotherapy. For example, anti-PD-1 antibody has been fused with IL-2, IL-15, or IL-21 to expand functional T cells and result in evident tumor remission [73, 130, 191]. Fused protein PD1-IL-2v bound PD-1 and IL-2βγ in cis on the same cell, enabled T cells to differentiate into stem-like cells and effector cells, alleviated terminally differentiated T_EX_ formation, and exhibited superior efficacy in the mouse pancreatic adenocarcinoma models [73]. Engineered αPD-1-IL-15R protein, fused anti-PD-1 antibody with IL-15-IL-15Rα, navigated sIL-15 to intra-tumoral T cells via cis-delivery [191]. It not only significantly expanded tumor-specific CD8(+) T cells for tumor rejection but also reduced systematic toxicity by concealing activity region with immunoglobulin Fc region [191]. In addition, the fusion protein PD-1Ab21 successfully reinvigorated tumor-specific T cells and promoted T_SCM_ proliferation [130].

## Conclusion

**Fig. 4:**
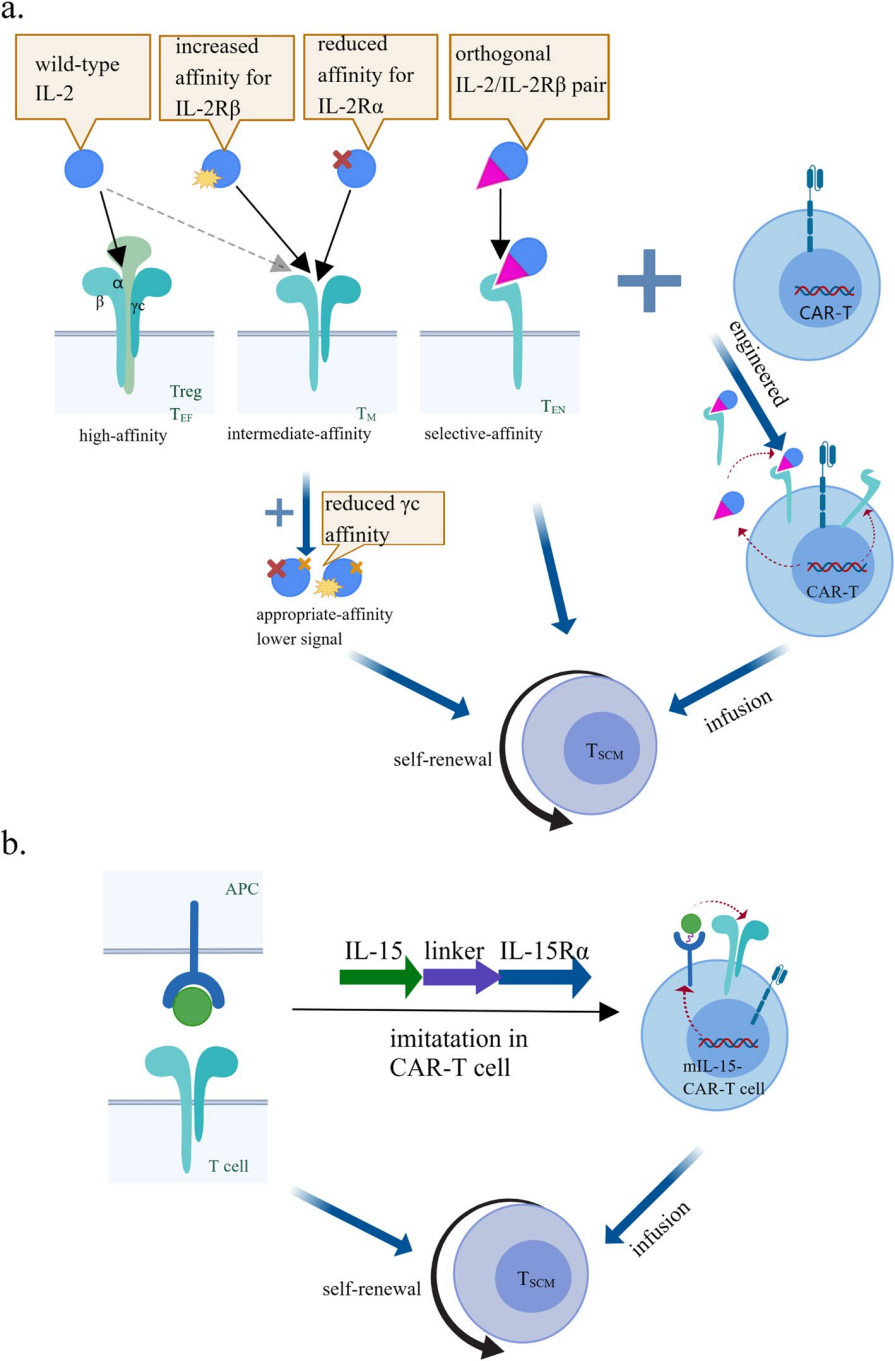
The engineered IL-2/IL-2R or IL-15/IL-15R for adoptively T_SCM_ induction. **A** Engineered IL-2 for selectivity to dimeric intermediate-affinity IL-2R and T_SCM_ induction. Activated T cells with high-affinity IL-2R finally go towards apoptosis by IL-2, and Treg with constitutively IL-2Rα deprive T cells of IL-2 to impair cytotoxicity. Reducing the affinity of IL-2 for IL-2Rα or increasing affinity for IL-2Rβ could target dimeric intermediate-affinity IL-2R and generate an appropriate affinity for T_SCM_ induction by further lowering the signal level. Applying to CAR-T engineering, engineered orthogonal IL-2 and IL-2Rβ system or fused protein will be expressed to function CAR-T cells to induce T_SCM_. **B** Engineered mIL-15 armored CAR-T cell for stemness maintenance. The trans-presentation of IL-15 by IL-15Ra on APC to T cells contributes to T_SCM_ induction independent of antigen stimulation. As a result, co-expressing IL-15 and IL-15Ra phenocopy the special presentation by a linker, yielding membrane-bounding IL-15. The mIL-15- CAR-T cells are able to differentiate into T_SCM_ in vivo

T_SCM_ belongs to a unique memory phenotype between naïve T cells and Tcm cells, possessing both stem-like memory and naïve phenotypic characteristics. Meanwhile, the number and proliferative capacity of T_SCM_ in the TME have gradually become valuable predictors of reactivity in tumor immunotherapy. A robust antitumoral immunity is dependent on the activation of immune cells, including CD8(+) T cells and CD4(+) T cells. Both cell types are activated by antigen presentation and play crucial roles in antitumor immune responses, particularly CD8(+) T cells. CD8(+) effector T cells, cytotoxic lymphocytes, serve as dominant killers of tumor cells directly, making the stemness of CD8(+) T cells an area of increasing interest. Theoretically, the more antigen-specific T_SCM_ in the TME, the stronger and more durable antitumor responses occur. Limited to the few intrinsic T_SCM_in vivo, the pursuit of inducing more “everlasting” T_SCM_ from naïve or effector T cells has gradually become a crucial goals of tumor immunotherapy research. At this point, it has been elucidated that the four γc family cytokines, including IL-2, IL-7, IL-15, and IL-21, could regulate the fate of T cell differentiation into T_SCM_ after antigen stimulation, and different combinations could make a different impact on T_SCM_ production and amplification. However, systemic utilization of these cytokines could bring some side effects, such as non-specific inflammatory toxicities, cytokine release syndrome, and off-target adverse. For example, high-dose IL-2 has been known to induce fatal capillary leak syndrome [192], while IL-15 has been associated with hypotension, thrombocytopenia, and other adverse effects included [193]. To further achieve high-quality and quantity T_SCM_ generation to maximize the antitumor effect and minimize the adverse effects, the development of gene editing techniques facilitates the production of multifunctional engineered T cells and optimal structure of the four γc family cytokines and their receptors [10, 76, 116, 123, 130]. Engineered versions have been developed to realize their full potential to promote the formation and persistence of T_SCM_ and to reduce efficacy-independent toxicities (Fig. 4) (Table 2). Furthermore, their delivery into the TME by engineering synthetic gene circuits on engineered T cells also lowers systemic toxicities and precisely controls infused T cell function [74, 80]. Despite their great potential for stemness maintenance, the immunogenicity of engineered proteins is a concern. Many clinical trials are currently conducted to search for an efficient protocol of the four cytokines for T_SCM_ formation and maintenance during ACT (Table 1). In addition to regulating T cell functions, they also exert an influence on other immune cells mainly dependent on the various expressions of their receptors, which play a pivotal role in different aspects of immunity. For example, IL-15 also promotes the proliferation and survival of NK cells, NKT cells, and mucosal associated invariant T (MAIT) cells that express IL-15R [193–196], and enhances the efficacy of NK cell associated transfer therapy [197, 198].

**Table 2:** Recently reported γc family cytokines or responding receptor engineered for transferred T cells stemness

Publication	Year		γc family cytokines	Engineered version	Discovery
Mo F et al. [76]	2021		IL-2	H9T, an engineered IL-2 partial agonist.	The H9T sustained the stemness of TCR-T and CAR-T cells through altered STAT5 signaling, mediating robust antitumor activity in vivo.
Aspuria PJ.et al. [79]	2021		IL-2	Orthogonal IL-2/IL-2Rβ pair, an orthogonal human IL-2 (STK-009) selectively pairs with an orthogonal human IL-2Rβ (hoRb) expressed on CAR T cells.	STK-009 expanded hoRb-expressing CAR T cells and maintained the presence of T_SCM_.
Kalbasi A et al. [10]	2022		IL-2 and IL-9 receptors	Orthogonal IL-2-O9R pair, IL-2Rβ-ECD-IL-9R-ICD (o9R), the chimeric receptor that orthogonal IL-2 receptor extracellular domain was fused with the intercellular domain of IL-9.	Co-culture of o9R-transduced pmel TCR-T cells with specific oIL-2 upregulated T cell stemness genes resulted in fantastic tumor eradication for ACT.
Hurton LV et al. [116]	2016		IL-15	mbIL-15, the mbIL15-CAR T cells co-expressing CAR with a membrane-bound chimeric IL-15 to incorporate the costimulatory properties of IL-15.	The mbIL-15 instructed CAR-T cells to possess a memory stem cell-like transcriptional profile and developed stemness attributes.
Guo J. et al. [123]	2021		IL-15	pro-IL-15, a next-generation IL-15 with the extracellular domain of IL-15Rβ fused to the N-terminus of super-IL-15-Fc (IL-15 fused with the IL-15Rα sushi domain) through a tumor-enriched Matrix Metalloproteinase (MMP) cleavable peptide linker to block its activity.	The pro-IL-15 specifically promoted stem-like CD8(+) T cells including infused T cells inside the TME making it possible to expand and persist of infused T_SCM_ in TME.
Li Y. et al. [130]	2021		IL-21	The fusion protein PD-1Ab21 by fusion of IL-21 to anti-PD-1antibody can be targeted to tumor-reactive T cells.	PD-1Ab21 promoted T_SCM_ generation and showed impressive antitumor effects.

After infusion, self-renewed T_SCM_ rapidly differentiated into effector T cells, yet the latter unavoidably experienced exhaustion and apoptosis in the immunosuppressive TME, another obstacle for sustained antitumor responses. Thus, robust and durable immune responses in vivo require not only sufficient resources of T_SCM_ cells to differentiate into better effector T cells efficiently, but also avoid infused T cell exhaustion after infusion for ACT. Administration of T cell growth factor IL-2 and PD-1/PD-L1 blockade may achieve the dual purpose of modifying the T cell exhaustion program and yield better effector T cells from T_SCM_ cells after the transfer, resulting in complete tumor remission [73, 190]. The underlying mechanism of synergy is that IL-2 stimulates antigen-specific T cells with stemness to differentiate into effectors and expand intensely, and PD-1 blockade inhibits exhaustion and improves the antitumor capacity. Similarly, IL-15 and IL-21 were designed to be fused with the anti-PD-1 antibody to yield fusion proteins αPD-1-IL-15R and PD-1Ab21, respectively, for tumor rejection [130, 191]. To our knowledge, the application of T_SCM_ has a bright future, as well as the orchestration of four γc family cytokines on adoptively transfer T_SCM_ cells in ACT, but the road is long and difficult.

## Data Availability

Not applicable.

## Abbreviations

ACT: Adoptive cell therapy
CAR: Chimeric antigen receptor
TME: Tumor microenvironment
T_SCM_: Stem cell-like memory T cells/stem cell memory T cells
Tcm: Central memory T cells
γc: The common cytokine receptor γ chain
IL-2/7/15/21: Interleukin-2/7/15/21
PD-1/PD-L1: Programmed cell death protein 1/ligand 1
ICB: Immune checkpoint blockage
CTL: Cytotoxic T lymphocyte
AICD: Activation induced cell death
NSCLC: Non-small cell lung cancer
TILs: Tumor infiltering T lymphocytes
GVHD: Graft versus host disease
GSK-3β: Glycogen synthase kinase-3β
Treg: Regulatory T cell
CD62L: CD62 ligand
AQP9: Aquaporin 9
TAG: Triglyceride
EOMES: Eomesodermin
GATA3: GATA Binding Protein 3
FAO: Fatty acid oxidation
LDH: Lactate dehydrogenase
mTORC1: mammalian target of rapamycin complex1
